# A Single Concussion in Juvenile Mice Leads to Sex Specific Acute Cerebral Vascular Dysfunction and Blood-brain Border Dysfunction

**DOI:** 10.21203/rs.3.rs-8622019/v1

**Published:** 2026-02-11

**Authors:** Jiamin Yan, Nathan Nguyen, Terese Garcia, Adam Godzik, Greer Cisneros, Amandine Jullienne, Junuen Alvarado, Rojina Pad, Jerome Badaut, Andre Obenaus

**Affiliations:** University of California, Riverside; University of California, Riverside; University of California, Riverside; University of California, Riverside; University of California, Riverside; University of California, Riverside; University of California, Riverside; University of California, Riverside; Centre d’Études Biologiques de Chizé; University of California, Riverside

**Keywords:** vessels, magnetic resonance imaging, closed head injury, vessel painting, Evans Blue

## Abstract

**Background:**

Traumatic brain injury (TBI) can induce alterations to the blood–brain border (BBB) that contributes to long-term neurological and behavioral deficits. The temporal progression of post-concussion BBB dysfunction during developmentally sensitive periods remains poorly understood. Therefore, we sought to characterize the temporal evolution of BBB disruption and cerebrovascular alterations acutely after concussion in juvenile mice.

**Methods:**

Postnatal day 17 (PND17) C57BL/6J male and female mice were subjected to sham or single closed head injury with long-term disorders (CHILD). At 1h, 6h, 1d, 3d, and 7d post-injury, Evans blue (EB) dye was administered intravenously to evaluate BBB permeability, followed by vessel painting to visualize modified cerebrovascular angioarchitecture. MRI-based T2 relaxation mapping at 1dpi has been used for brain tissue properties, including edema. EB and vascular features were modeled to assess ability to discriminate between sham and CHI mice.

**Results:**

A single early-life concussion induced hyper-acute (hours) structural and functional alterations in brain vasculature. CHILD in PND17 mice resulted in: 1) disruption of physiological functions and developmental trajectories, 2) reduced brain volumes and sex-dependent T2 relaxometry changes (elevated in females, reductions in males), and 3) hyper-acute BBB increases in permeability which correlated with cerebral vascular rarefaction. Notably, males exhibited more robust BBB and vascular perturbations than females, revealing sex-dependent trajectories of vascular response to CHILD. We also highlight differential vulnerability in vessel location with the smaller penetrating cortical vessels displaying greater susceptibility to alterations compared to larger, more resilient pial blood vessels. Modeling demonstrated that vascular features clustered together while trajectory analysis confirmed that female CHI mice were not consistent in their disease trajectory compared to male CHI. Additional analysis suggested that vascular features able to discriminate in a sex- and injury specific manner.

**Conclusions:**

A single concussion is sufficient to induce hyper-acute BBB and cerebrovascular perturbations in juvenile mice, which may presage long-term deficits during development. Importantly, sex differences in vascular TBI responses evident at PND17 emphasize the need to consider sex as an important variable in future pediatric TBI research.

## Background

Traumatic brain injury (TBI) due to external mechanical force, represents a major public health and economic burden, accounting for more than 600,000 emergency department visits each year, with 90% of all pediatric TBIs classified as mild TBI (mTBI) [1]. Little is known about the unique features of mTBI in children, particularly considering the structural and functional differences as the developing brains transition to adulthood. Thinner and less rigid skulls may provide reduced mechanical protection, increasing susceptibility to fractures and tissue deformation [2, 3]. The developing brain is characterized by immature neural networks and active processes of synapse formation and pruning, such that perturbations during these critical epochs can interfere with normative maturation [4]. Pediatric and juvenile brain injury places children at elevated risk for persistent learning disabilities, psychological disorders, behavioral problems, disruption of academic and social performance [1, 5, 6].

The blood-brain barrier or more recently designated as Blood-Brain Border (BBB) interface [7], is a very dynamic interface between blood and brain, composed of tight junction proteins linked endothelial cells expressing various transporters finely tuned by pericytes and astrocytic endfeet [7]. BBB remodeling and vascular dysfunctions have been suggested to contribute to the long-term neurological and behavioral deficits often observed after adult TBI [8, 9]. Recent work in patients with moderate to severe TBI exhibited BBB perturbations confined to microvascular regions in pediatric TBI but was predominately in larger vessels in adult patients [10]. Moreover, increased BBB dysfunctions in juvenile mice evoked an increased microglial response [11]. Yet, whether similar mechanisms occur following juvenile mTBI, and, if so, the timeline and severity of these alterations in the developing brain are underexplored.

Most children appear to recover from mTBI within several weeks but up to one-third experience persistent deficits [1]. In adults, BBB dysfunction is recognized as a central mechanism contributing to long-term dysfunction after severe TBI in humans [12, 13] and in adult rats exposed to severe TBI [13]. While moderate to severe TBI outcomes have been relatively well documented in both human subjects and in rodent models, far less is known about the sequelae following mTBI/concussion in pediatric brain injury. Indeed, the temporal course of hyperacute BBB changes in pediatric and juvenile mTBI have not been reported. This lack of mechanistic understanding impedes the development of pediatric-specific diagnostic tools, treatments, and strategies to identify children at greatest risk for chronic deficits.

To address this gap, we examined BBB integrity and altered angioarchitecture in a juvenile closed head injury with long-term disorders (CHILD) model [14]. The CHILD model is a robust unrestrained closed head concussion model in postnatal day 17 (PND17) mice and replicates key clinical features of mTBI: a) rotational acceleration and coup–contrecoup injury [14, 15], b) behavioral alterations [15–17], c) acute perturbations in tissue oxygenation, neurovascular coupling and long-term cardiac dysfunction [16, 18], and d) progressive decrements in white matter [19] (see Table 1 in reference [19]). In our study, we utilized the PND17 CHILD model and examined BBB leakage and cerebrovascular perturbations at 1h, 6h, 1-, 3- and 7-days post-injury (dpi) in a sex-specific manner. We report both temporal and sex-specific alterations in BBB and vascular responses to juvenile mTBI.

## Methods

**Figure 1:**
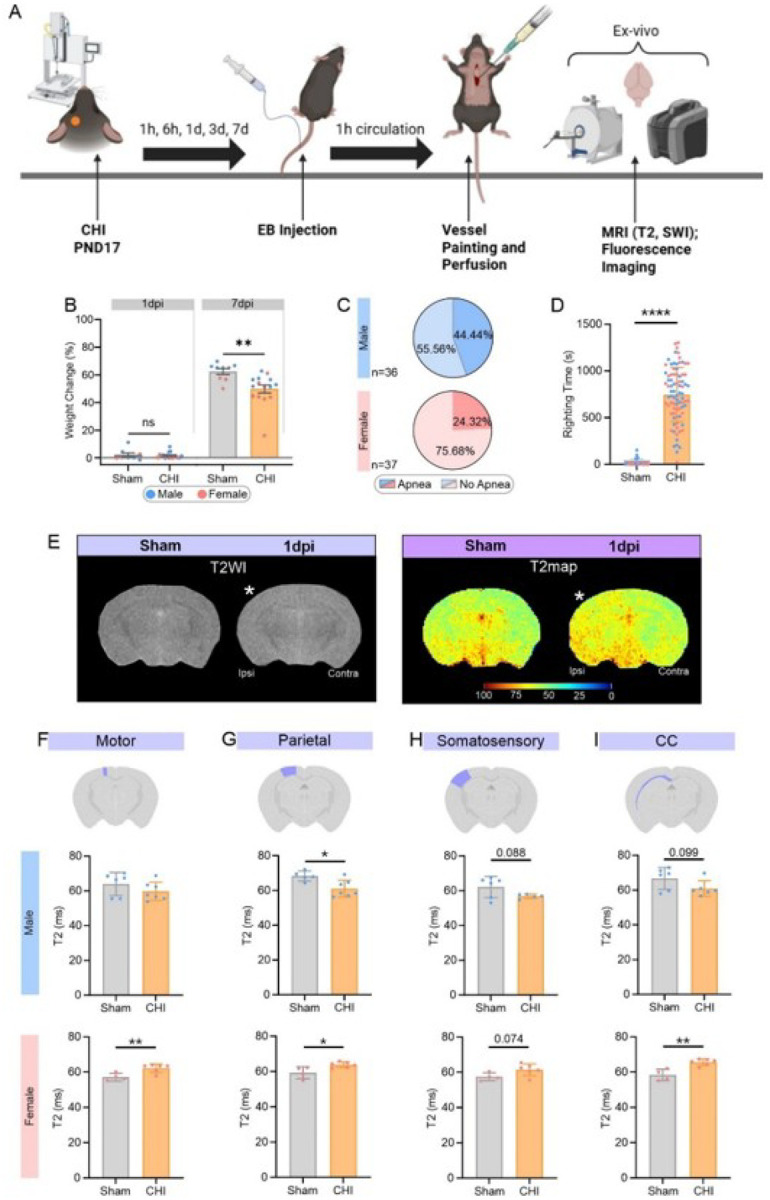
Sex differences emerge in temporal characterization of CHILD. **(A)** Schematic of experimental design and timeline. **(B)** Body weight relative to pre-CHI weights were not different at 1dpi but were significantly reduced at 7dpi compared to shams. (*p ≤0.05, unpaired t-test).**(C)** Pie charts report that 44.44% of males had apnea compared to 24.32% of females after CHILD induction. **(D)** Time to right was significantly increased after CHILD compared to shams (****p≤0.0001, unpaired t-test). **(E)**Representative MRI T2-weighted images (T2WI) and T2 maps of sham and CHILD brains at 1dpi (*denotes the injury). **(F-I)** Ipsilateral T2 relaxation times (ms) were used to assess edema. **(F)** T2 relaxation times in the motor cortex were significantly increased in females (**p≤0.01, unpaired t-test) but not in males relative to shams. **(G)** T2 values in parietal cortex were significantly increased in both males and females (*p≤0.05, unpaired t-test). **(H)**T2 relaxation (ms) did not differ significantly but were increased compared to shams in the ipsilateral somatosensory cortex from males and females. (**I)**T2 in the ipsilateral corpus callosum (CC) was significantly increased in females (**p≤0.01, unpaired t-test) but not in males **(J)**. All Bar graphs were represented as mean ±SD.

The experimental protocol focused on hyperacute and acute time points after mTBI as outlined in a schematic in Fig. 1A.

### Animals

Pregnant C57BL/6J female mice (E14) were purchased from Jackson Laboratory (JAX #000664). CHILD or sham procedures were performed on postnatal day 17 (PND17) pups of both sexes. Animals were randomly assigned to one of six groups (Supplemental Table 1): Sham, CHILD 1h (n = 13), CHILD 6h (n = 16), CHILD 1d (n = 21), CHILD 3d (n = 18), and CHILD 7d (n = 19). Pups were excluded if their weight was less than 5.9g on PND 17; all pups were weaned on PND 21. Mice were maintained at 21°C with an automated 12-hour light-dark cycle and had *ad libitum* access to water and standard vivarium chow. All experiments were in accordance with the University of California, Riverside and University of California, Irvine Institutional Animal Care and Use Committees and federal regulations and in accordance with ARRIVE guidelines as well as Animal Welfare Act and Public Health Service policies related to humane care of animals.

### Closed Head Injury with Long Term Disorders (CHILD)

CHILD model details and videos have been published recently [14]. Briefly, on PND17, animals were weighed and anesthetized with 2.5% isoflurane in 1.5 L/min O_2_ for 5 minutes in a chamber heated to 37°C. Each mouse was removed from the isoflurane chamber and quickly placed on a taut and secured aluminum foil (15 × 15 cm) stretched across a stereotactic frame. The mouse position was adjusted so that the impactor tip was directly above the left somatosensory cortex. The impactor tip (3mm diameter rubber tip) was mounted at a 90° angle perpendicular to the stereotactic apparatus. A single impact was then delivered using an electromagnetic impactor (Leica Biosystems, Deer Park, IL, USA) with the following parameters: velocity: 3m/s, dwell time: 0.1s, and depth: 3mm. The resulting injury is equivalent to Grade 2 (G2) level injury, as previously defined [15]. The presence of apnea and head rotation were recorded. The mouse was then immediately placed on its right side in a warmed (37°C) recovery chamber to assess righting time and time to resume exploratory behaviors. All animals survived the CHILD. The shams underwent identical procedures but without an impact.

### Evans Blue Injection, Vessel Painting and Tissue Fixation

At each time point post-CHILD, a 2% solution of Evan’s Blue (EB) (Acros Organics, Geel, Antwerpen, Belgium) in phosphate buffered saline (PBS) was administered via tail vein injection (3μL/g) while the animals were under light anesthesia (2% isoflurane in 1.5L/min O_2_). EB was allowed to circulate for 1 hour prior to vessel painting and perfusion. Mice were then anesthetized with 2.5% isoflurane in 1.5L/min oxygen and were given an intraperitoneal (i.p.) injection of Ketamine (200mg/kg) and Xylazine (200mg/kg) to induce deep general anesthesia. Mice were then given an i.p. injection of heparin (1000units/kg) followed by sodium nitroprusside (0.75mg/kg) to dilate vessels. To visualize the cerebrovasculature, we performed an intracardiac injection of 3,3’-dioctadecyloxacarbocyanine (DiO, Biotium, Fremont, CA, USA) (0.75mg/kg) diluted with 4% dextrose in PBS. Mice were then immediately intracardially perfused with 15mL PBS followed by 20mL of 4% paraformaldehyde (PFA). Brain tissues were post-fixed in 4% PFA for 24h, washed with PBS for 3 consecutive days and stored at 4°C in 0.02% sodium azide-PBS solution. Labeling of the vasculature is termed vessel painting (VP) [20].

### IgG Immunohistochemistry (IHC) and Analysis

1hpi mouse brains were used for IgG staining and were incubated in 30% sucrose solution at 4°C for 48hrs. Samples were then frozen in Optimal Cutting Temperature Compound (OCT) on dry ice and stored at −20°C. Brain samples were sectioned coronally into 30μm thick slices and mounted directly onto slides and stored at −80°C. Sections were treated with 1% Sodium Dodecyl Sulfate at room temperature then incubated for 1.5 hours in room temperature with Alexa Fluor^™^ 594 Goat anti-Mouse IgG (1:1000, Invitrogen, A11005). Slides were then dried and coverslipped with Vectashield mounting medium with DAPI (Vector Laboratories, Burlingame, CA, USA).

### Wide-field and Confocal Microscopy

Fluorescence images from vessel painted brains were acquired with a wide-field fluorescence microscope (Keyence BZ-X810, Keyence Corp, Osaka, Japan). Both axial surface and coronal sections at the level of the dorsal hippocampus (Bregma − 1.82mm) were imaged at 2X using the sectioning and Z-stack functions (step size 25.2 μm, 20 stack). Level correction, black balance, and haze reduction (blur size = 10, brightness = 10, reduction size = 1) were applied to the images using BZ-II Analyzer software (Version: 1.1.30.19). Higher magnification 10X images were taken from regions with EB extravasation and the corresponding region in the contralateral hemisphere.

Confocal images at 20X were acquired from 30μm IgG-stained sections using a Zeiss LSM 880 confocal microscope (Carl Zeiss, Oberkochen, Germany). IgG, EB, and VP signals were imaged using excitation wavelengths of 561, 633, and 488 nm, respectively. Single-field images were acquired using the following parameters: 2% laser power; 2.57 Airy units pinhole size; 25 optical sections of z-stack with a step size of 2 μm; and 425.1 μm × 425.1 μm image field. Three-dimensional reconstruction and visualization were performed using Imaris Bitplane software (version 10.2.0; Oxford Instruments, Abingdon, UK).

### Evans Blue Analysis

Quantification of EB extravasation was performed using Fiji (Version: 1.54f) software. First, a known region of EB leakage was outlined in a single CHILD mouse at 1hr post injury. Then the “fire” lookup table was applied and intensity levels > 100 were defined as extravasation. This method was then applied to all mice and regional areas with intensity values > 100 was extracted and summarized in MS Excel. In coronal sections, the integrated density was measured within the identical cortical region of the ipsilateral hemisphere corresponding to the site of the injury in all CHILD and sham mice.

### Angioarchitecture Analysis

Angiotool 0.6 software [21] was used to quantify classical vessel characteristics (vessel density, length, and junction density in the selected region of interest (ROI). Vessel complexity was assessed using the ImageJ FracLac to derive local fractal dimensions [22]. Axial regions of interest (ROI) included left and right hemispheres or whole axial brain analyses. Coronal ROIs encompassed cortical regions extending from the mid-line to the ventral-most boundary of the somatosensory cortex and placed ipsi- and contralaterally. Similar analytical methods have been published previously [23]. Data was extracted and summarized in MS Excel.

### Magnetic Resonance Imaging (MRI) Acquisition and Analysis

T2-weighted (T2WI) and susceptibility-weighted imaging (SWI) were performed on *ex vivo*, skull-attached samples at 9.4T (Bruker Biospec, Billerica, MA). The following acquisition parameters were used for T2: 4000ms repetition time, 10ms echo time, 10 echoes, 4 averages, field of view 1.25 × 1.25cm, matrix 128 ×128, 20 slices, 0.5mm slice thickness, 0.5mm slice interval, acquisition time ~ 25min using Paravision 5.11. SWI was acquired using: 722.9ms repetition time, 10ms echo time, 8 averages, field of view 1.25 × 1.25cm, matrix 128 ×128, 20 slices, 0.5mm slice thickness, 0.5mm slice interval, acquisition time ~ 12min.

The brain was segmented away from skull and extraneous tissues using ITK_SNAP (Version 3.8.0) software [24]. The extracted brains were used to generate T2 maps using JIM 7.0 software (Version. 7.0_42 Jan 10 2018, Xinapse Systems, Northants, UK). T2 maps were registered to our modified bilateral Australian Mouse Brain Mapping Consortium Atlas [25] using Advanced Normalization Tools (ANTS, Version:RRID:SCR_004757, University of Pennsylvania, Philadelphia, USA). Regional brain volumes and T2 relaxation times were then derived from the registered T2 maps. SWI scans were analyzed using Signal Processing in NMR (SPIN) software (Version: Revision 1872) to identify presence of extra parenchymal bleeds.

### Modeling Methodology

All the data, except MRI, were combined for the analysis to determine if there were potential predictors for CHI BBB disruption. The analysis was performed by the in-house python scripts using SciPy and Scikit-learn libraries. MRI data from a sub-cohort of mice these data were excluded, as they were missing over 70% of samples. Imputation. Up to 17% of data in other feature groups were missing; these were imputed to allow consistent downstream statistical analysis and module construction (see below). Imputation was performed separately for each variable using a hierarchical strategy, as follows. If at least two real observations were available within the same group × sex × timepoint subset, the missing values in that subset were replaced with the mean of the available observations. If a subset contained fewer than two real measurements (i.e., insufficient information for a reliable subgroup mean), the missing values were left unchanged and only replaced with the global mean of that variable if still required for principal component analysis (PCA) or visualization. This approach preserved true biological variability, avoided overfitting sparse subgroups, and prevented downstream analyses (e.g., PCA, clustering) from being dominated by “missingness” patterns rather than biological signal.

Heatmap and Clustering. To visualize the correlation structure among measurements and assess relationships between features, pairwise Spearman correlation coefficients were computed for all features across all animals. Correlations were displayed as a heatmap with hierarchical clustering using average linkage and a Euclidean distance metric on the correlation matrix. This unsupervised approach recovered biologically related variables and highlights modules of coordinated change following CHI, supporting intuition that measurements from the same anatomical orientation or imaging modality are correlated with each other. Module Definitions. Clustering results were used to define feature “modules” that both describe related biological processes and are correlated with each other. For example, vascular metrics derived from coronal sections formed a vascular-coronal module, while fractal dimension (LFD) features from axial slices defined as an axial-LFD module. For each module, we used PCA and used the first principal component (PC1) of the standardized module variables to be used as the module’s composite metric. PC1 captures the dominant shared variance of the module and serves as a noisereduced, direction-consistent representation of the underlying biological process, such as vascular remodeling or vascular complexity.

Trajectory Modeling. We took a combined p-values approach for trajectory modeling (averaging over points) to assess changes over time. To quantify sex differences while properly accounting for measurements collected at multiple timepoints after injury, statistical comparisons were performed independently at each timepoint using the Mann–Whitney U test. The resulting per-timepoint p-values (p_i_) were then aggregated into a test statistics (**Ψ**) using Fisher’s combined probability method, as defined:

$$\psi =-2\sum_{i=1}^{k}\text{ln}\left(p_{i}\right)$$

which follows a c^2^ distribution with 2k degrees of freedom (k = number of timepoints) and allows us to calculate the combined p-value. This approach does not assume linear or monotonic changes over time and is robust to heterogeneous variance and missingness across timepoints. For additional robustness, permutation-based combined p-values were computed by shuffling sex labels within each timepoint, recomputing p-values, and comparing the observed Fisher statistic to its permutation distribution. Consistency. Because Fisher’s method combines p-values but not effect directions, we also quantified whether the male–female differences were directionally consistent across timepoints. For each timepoint, the sign of the difference (mean_male − mean_female) was recorded. Directional consistency was defined as the fraction of time points at which the sign matched the majority direction across the trajectory. Values near 1.0 indicate stable directional effects (e.g., males consistently higher than females), whereas values near 0.5 indicate mixed or fluctuating differences. This provides an intuitive measure of biological coherence complementing the combined p-value.

t-SNE Embedding of Key Discriminative Features. To visualize multivariate relationships among subjects, we applied t-distributed Stochastic Neighbor Embedding (t-SNE) to a curated feature set consisting of the most biologically discriminative module PC1 scores (e.g., axial LFD, coronal vascular) and key volumetric variables. All features were standardized prior to embedding. Only animals with complete data for the selected features were included, ensuring stable geometry and avoiding distortions driven by missing values. The resulting two-dimensional embedding was plotted with point color indicating sex, point shape indicating Sham or CHI groups, and where small numeric labels marking post-injury timepoints. This provides an intuitive visualization of how injury and sex jointly influence high-dimensional phenotypic space.

### Statistical Analysis

Statistical analysis was performed using GraphPad Prism (Version 9, GraphPad, Boston, MA, USA). We performed one-way analysis of variance (one-way ANOVA) with multiple comparisons for temporal data and group comparisons utilized t-tests. All t-tests were parametric unless specifically stated. Pearson correlations were also performed in GraphPad. All values are presented as mean ± SEM. Statistical significance threshold was defined as p < 0.05 with trending reported in those cases with p < 0.10.

## Results

### CHILD induced sex-specific physiological and structural changes.

Prior to CHILD induction, PND17 weights between male and female mice were not significantly different, with the average weight of all pups being 7.04 ± 0.67g (n = 117). There were no significant differences (p = 0.724, unpaired t-test) between male average weights (7.06 ± 0.59g, n = 61) and female weights (7.02 ± 0.75, n = 56). No significant weight differences were found at 1dpi between sham and CHILD male or female mice (Fig. 1B). In contrast, relative to pre-CHILD (baseline), weight gain at the 7dpi period relative was significantly increased in sham compared to CHILD mice (p = 0.0009, unpaired non-parametric t-test) (Fig. 1D). Male CHILD mice at 7dpi had a significant decrement (p = 0.002, unpaired non-parametric t-test) in weight gain (55.6 ± 0.04% compared to male shams 66.5 ± 0.02%; Supplemental Fig. 1A). Female CHILD mice also had reduced weight gain compared to female sham mice at 7dpi but did not reach significance (p = 0.073, unpaired non-parametric t-test) (Supplemental Fig. 1B). Paired-weight changes between baseline and 1dpi or 7dpi further demonstrate significant increases in weight gain over the 7dpi period (Supplemental Fig. 1C, D).

Immediately after CHILD induction we monitored the level of consciousness in all mice by recording the presence and duration of apnea immediately after head impact and the time required to resume a righting position. The prevalence of CHILD mice that exhibited apnea was ~ 20% higher in males (44.44%) than in females (24.32%) (Fig. 1C), exhibiting a sex-specific immediate physiological response to concussive injury. CHILD mice also exhibited a significantly longer time to resume righting position relative to shams (p < 0.0001, unpaired t test) with no overt sex differences (Fig. 1D).

A randomized subset of sham (n = 11) and CHILD mice (n = 15) at 1dpi underwent *ex vivo* T2-weighted MRI (Fig. 1E). Cerebrum volumes exhibited sex differences with male CHILD mice showing significantly reductions by 8.82% (p = 0.035, unpaired t test) compared to shams (Supplemental Fig. 2A), while female CHILD or sham mice did not report differences. Male CHILD mice exhibited significantly lower cerebrum volumes than female CHILD mice (p = 0.011, unpaired t test) but no differences between male and female shams were reported (Supplemental Fig. 2A). Brain tissue properties were assessed with T2-relaxometry measurements (in ms) from cortical regions (motor, parietal and somatosensory) and white matter structures (corpus callosum, CC) that are at the site of the concussive impact (Fig. 1F–I). T2 relaxation time was reduced in all four regions in male CHILD mice. There was a significant decrease in T2 relaxation in parietal cortex of male CHILD mice of 12.58% compared to shams (p = 0.014, unpaired t test) (Fig. 1G). Sex differences were also observed since female CHILD mice had significantly increased T2-values in motor (8.82%), parietal cortices (7.56%), and corpus callosum (10.21%) compared to female shams (p = 0.010, p = 0.021, p = 0.002, respectively, unpaired t test) and a trending significance in the somatosensory cortex (p = 0.074, unpaired t test) (Fig. 1F–I).

Susceptibility-weighted imaging (SWI) was also acquired to assess the presence of extravascular blood (Supplemental Fig. 2B) which was often found at the cortical surface and at the interface between gray and white matter (corpus callosum). Seventy-five percent of male CHILD mice but only 42% of female CHILD mice exhibited visible extravascular bleeding (Supplemental Fig. 2C). Thus, clinically relevant neuroimaging further confirms sex-specific differences in water content and parenchymal bleeds.

### CHILD induced transient dysfunction of the blood-brain border (BBB) at acute time points followed by recovery.

**Figure 2:**
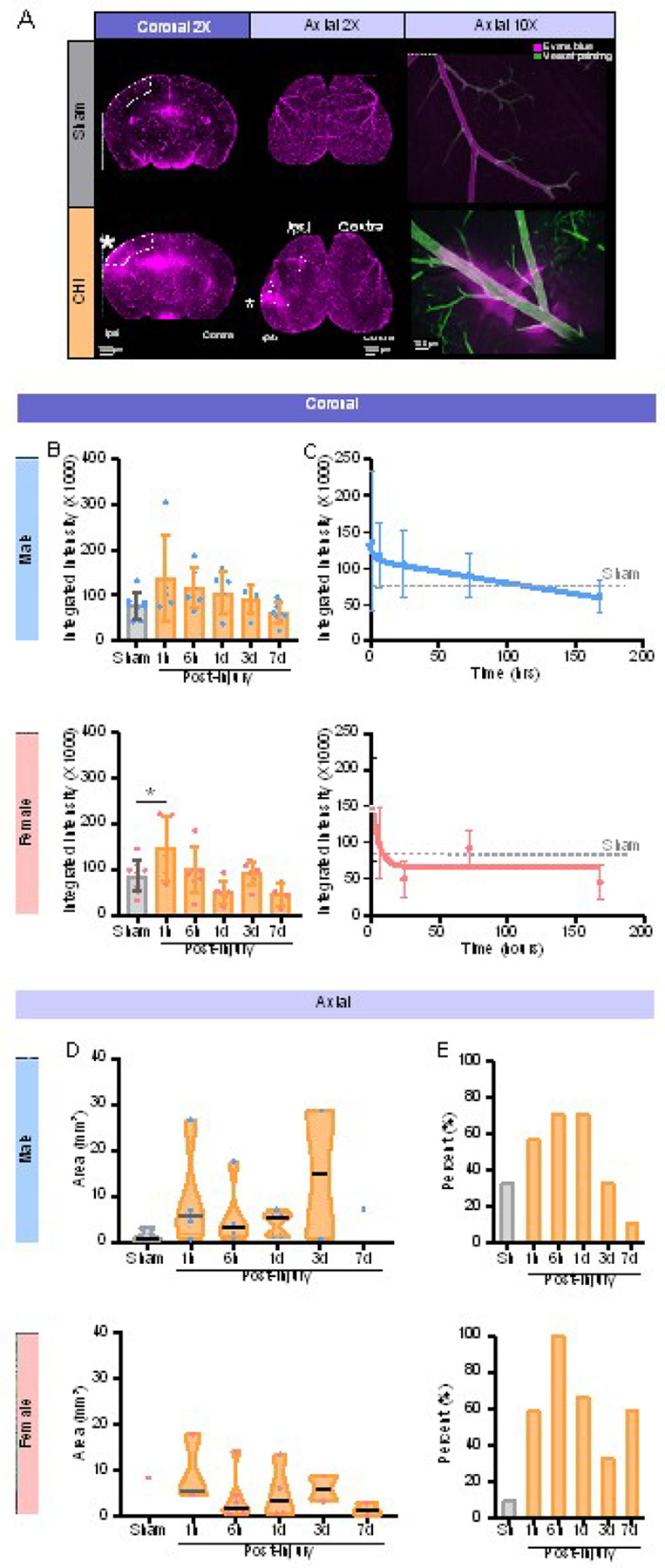
BBB disruption is most evident at hyper-acute periods following CHILD. **(A)** Representative 2X images of Evans Blue (EB) fluorescence from axial and coronal aspects of sham and CHILD brains (left) at 1hpi. White dotted outlines indicate the quantification regions.10X images of representative blood vessels from the axial surface of sham mice which exhibit no vascular leakage compared to CHI mice whose vessels exhibit EB extravasation. **(B)**Coronal EB integrated intensity from the lesion site on the ipsilateral cortex in males and females were elevated at 1hpi (significance in females p<0.05, one-way ANOVA) that slowly declined over the 7dpi timepoints. **(C)** Integrated density values across the experimental period were curve-fitted using a two-phase exponential decay model and revealed more linear leakage over time in males, while females exhibited a rapid exponential decrease in EB extravasation after a single CHI. **(D)** EB extravasation area was measured from the brain surface but while elevated no significant differences were observed between time points nor sex. **(E)** The percentage of male CHILD mice that exhibited BBB at each time point was elevated over the first 24hrs but then rapidly declined. Female mice showed a higher percentage of mice with EB leakage that was relatively sustained over the 7dpi experimental period. All bar graphs were represented as mean ±SD

Evans Blue (EB) is a water-soluble fluorescent dye that binds to serum albumin and only permeates into the brain parenchyma when BBB properties are compromised. EB observed within blood vessels confirmed functional perfusion in sham mice, whereas EB accumulation within the brain parenchyma was observed in the ipsilateral cortex in CHILD mice at 1hpi (Fig. 2A). At higher magnification (Fig. 2A, right panel), EB extravasation in the parenchyma was observed adjacent to vessels defined by VP at the site of injury in CHILD mice, but not in shams. Integrated intensity of extravasated EB in the parenchyma was quantified at 1hpi, 6hpi, 1dpi, 3dpi and 7dpi in the ipsilateral cortex from coronal tissue sections. Increased EB accumulation peaked at 1hpi and then declined over time in males and females (Fig. 2B). Female CHILD mice exhibited a significant increased EB extravasation of 71.02% compared to shams at 1hpi (p = 0.047, unpaired t test), while the increase of EB extravasation did not reach significance in male CHILD mice (Fig. 2B). Higher variability in EB extravasation was observed for the acute timepoints (1hpi and 6hpi) compared to later timepoints in both sexes.

Using a two-phase exponential decay model, temporal analysis of BBB dysfunction from coronal images showed a linear decrease in EB extravasation in males, while females exhibited a rapid exponential decrease after injury (Fig. 2C). We also assessed EB leakage area from the cortical surface (axial) (Fig. 2A, middle panel) and like the coronal analyses, there was considerable variability in male CHILD mice although less so in the female mice with no significant differences (Fig. 2D). The number of mice from each sex who had axial EB leakage present were collated as a percent of all the mice (Fig. 2E). In male CHILD mice there was an increasing proportion that showed BBB leak that peaked at 1dpi (71%) and then precipitously declined by 7dpi (11%) (Fig. 2E). In contrast, CHILD female mice had 100% of injured mice exhibiting cortical EB leakage at 6hpi that slowly declined by 7dpi (60% of mice, Fig. 2E). Exemplar micrographs illustrate extravascular EB extravasation from cortical vessels are shown in Supplementary Fig. 3, at 1hpi and 1dpi. These images reveal subtle and vascular localization of EB leakage within cortical regions and those adjacent to the concussive impact site. These findings were in line with sex differences in BBB pathophysiology between concussed male and female mice.

### CHILD impairs axial cortical angioarchitecture associated with BBB perturbations

**Figure 3:**
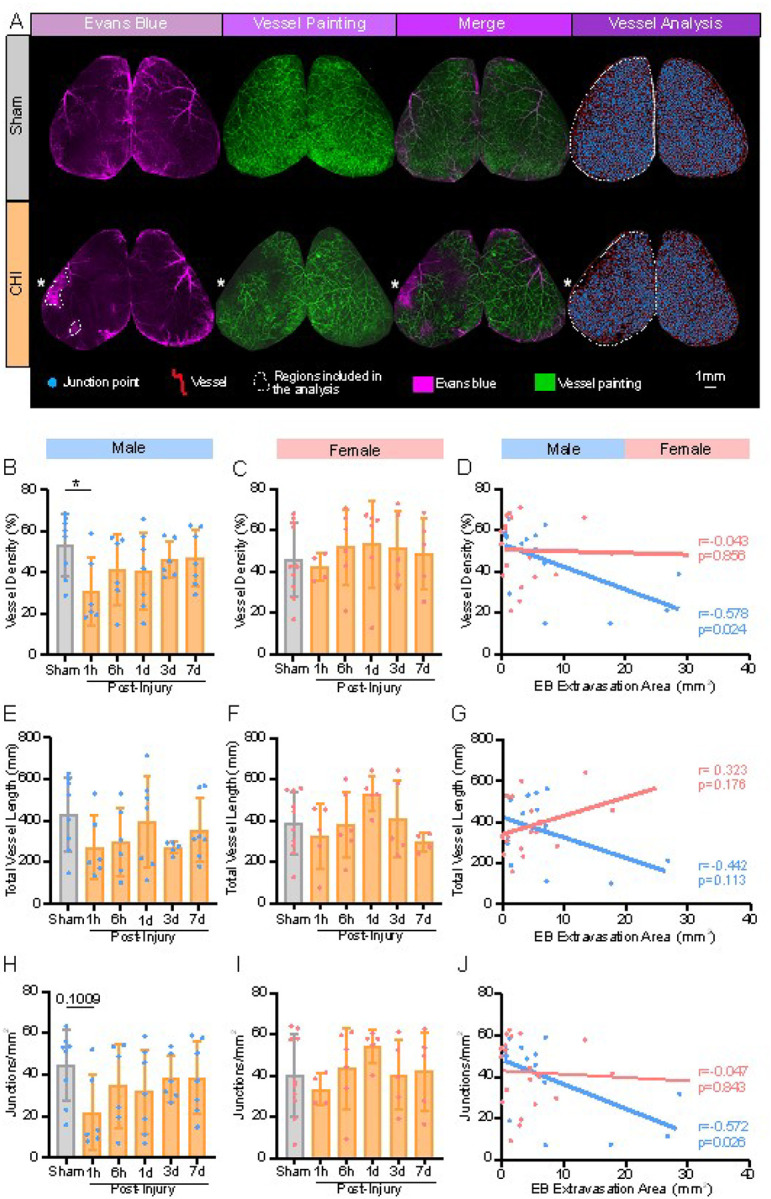
Acute vessel perturbations correlate with severity of Blood-Brain-Border (BBB) disruptions. **(A)** Representative axial and EB extravasation (magenta), cerebrovasculature (green), merged EB and VP, and vessel analysis maps illustrating junctions (blue dots) and vessels (red lines) in representative sham and CHILD mice. White dotted outlines denote regions of EB extravasation and axial surface analyzed. **(B)** Vessel density in CHILD males was significantly reduced at 1hpi compared to shams (*p≤0.05, one-way ANOVA). **(C)** No significant differences in vessel density were observed in females. **(D)** In males, EB extravasation area was significantly correlated with vessel density across all time points (Pearson’s r= −0.5781, *p≤0.05) but not in CHILD females. **(E-F)** Total vessel length in both CHILD males **(E)** and females **(F)** did not differ significantly from shams and yielded no significant correlations EB extravasation area and total vessel length **(G). (H)** Junction density at 1hpi exhibited a trending reduction in CHILD males compared to shams (p=0.1009, one-way ANOVA). **(I)** No significant alterations in junctions were reported in females. **(J)** In CHILD males, EB extravasation area was significantly correlated with junction density (Pearson’s r= −0.5723, *p≤0.05) but not in CHILD female mice. All bar graphs were represented as mean ±SD

The inter-relationships between axial cortical vascular features using vessel painting and EB extravasation were examined in sham and CHILD mice across all timepoints (Fig. 3A). Axial vessel density on the ipsilateral hemisphere was significantly reduced by 41.79% in male CHILD mice compared with shams at 1hpi (p = 0.047, one-way ANOVA), which progressively recovered by 7dpi (Fig. 3B). Total vessel lengths and number of junctions exhibited no significant changes in CHILD males, but the pattern of changes were like that of vessel density in males (Fig. 3B, E, H). However, no significant changes were observed in females either in vessel density, total vessel lengths and number of junctions at any of the time points examined (Fig. 3C).

In males the temporal resolution of vessel density was consistent with the peak BBB dysfunction (Fig. 2B, C). Therefore, the relationships between these outcome measures were calculated (Fig. 3D, G, J). In male CHILD mice axial surface EB extravasation area were significantly negatively correlated to vessel density (r=−0.578, p = 0.024) (Fig. 3D) and junction density (r=−0.572, p = 0.026) (Fig. 3J). CHILD male mice exhibited no significant relationship with total vessel length and surface EB extravasation (Fig. 3G). This suggests that vessel alterations characterized by a loss in density and number of junctions also demonstrated BBB dysfunction. No significant correlations were observed in CHILD females in any vessel metric compared to EB extravasation area (Fig. 3D, G, J). These results suggest that impairment of the BBB is strongly associated with morphological vessel alterations in males but not in females, highlighting sex differences in cerebrovascular pathophysiology early post-concussion.

### Coronal cortical angioarchitecture is decreased after CHILD and is associated with BBB perturbations

**Figure 4:**
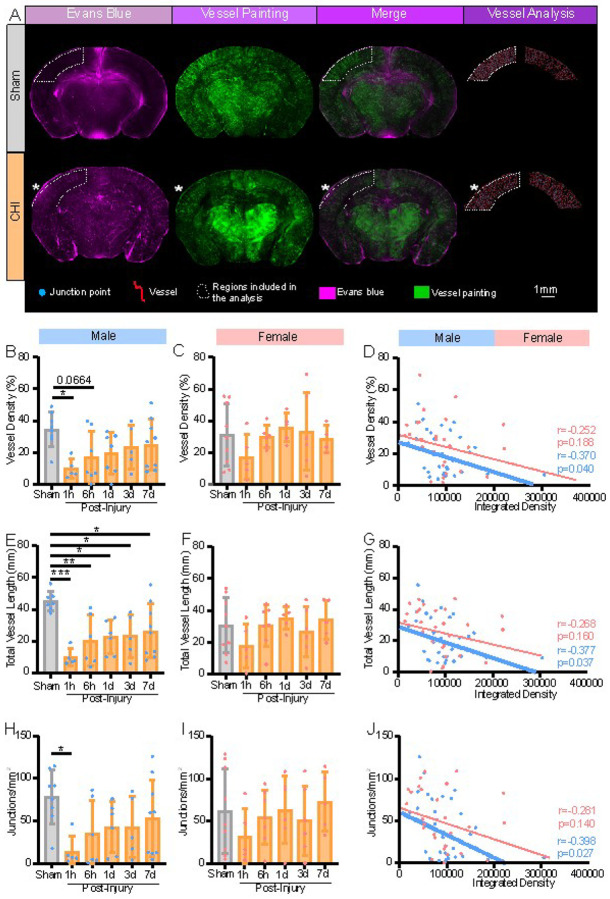
Cortical cerebrovascular alterations correlate with severity of BBB disruptions. **(A)** Representative coronal images of EB extravasation (magenta), vessel painting (green), merged EB and VP images, and derived cortical vessel analysis from sham and CHILD brains. White dotted outlines denoted regions of EB extravasation and coronal VP included in the analysis. **(B)** in CHILD males exhibited significant reductions in vessel density at 1hpi compared to shams (*p≤0.05, one-way ANOVA) with a trending reduction at 6hpi (p=0.0664, one-way ANOVA). **(C)** No significant vessel density differences were observed in females. **(D)** In CHILD males, EB extravasation (integrated density) in the injured cortex (* denotes CHI site) significantly correlated with vessel density (Pearson’s r= −0.3701, *p≤0.05) but not in females. **(E)** Total vessel length in CHILD males exhibited significant reductions at all time points (*p≤0.05, **p≤0.01, *** p≤0.001, one-way ANOVA). **(F)** No significant differences were observed in CHILD females. **(G)** In CHILD males EB extravasation at the lesion site significantly correlated with total vessel length (Pearson’s r= −0.3765, *p≤0.05) but not in females. **(H)** CHILD males reported a significant reduction in junction density at 1hpi compared to shams (*p≤0.05, one-way ANOVA). **(I)** No changes in junction density were found in females (p>0.05, one-way ANOVA). **(J)** EB extravasation in CHILD males significantly correlated with junction density (Pearson’s r= −0.3981 *p≤0.05) but not in females. All bar graphs were represented as mean ±SD.

The ipsilateral coronal cortical vasculature was analyzed at and adjacent to the impact site, examining the vessels penetrating the cortex. We quantified VP angioarchitecture and EB extravasation, similarly to the axial surface findings (Fig. 4A). Following similar pattern observed on axial analysis (Fig. 3B), a dramatic and significant reduction by 71.82% cortical vessel density was found in male CHILD mice at 1hpi compared to shams (p < 0.05, one-way ANOVA, Tukey’s post-hoc test), which temporally recovered by 7dpi (Fig. 4B). Total vessel length was significantly reduced at every time point in CHILD males (p < 0.05 for each, ordinary one-way ANOVA) (Fig. 4E). Junction density was similarly significantly reduced in CHILD male mice at 1hpi compared to shams (p = 0.05, ordinary one-way ANOVA) (Fig. 4H). As described for the axial analysis, no significant morphological changes in blood vessel metrics were observed in female CHILD mice compared to shams (Fig. 4C, F, I), despite an overall trend for vascular reductions at 1hpi.

When we examined the relationship between vascular morphological features (vessel density p = 0.040; total length p = 0.037; junctions p = 0.027), we found significant correlation to EB intensity in CHILD male mice independent of time post injury (Fig. 4D, G, J). Again, no significant correlations between EB extravasation and vessel metrics were found in female CHILD mice(Fig. 4D, G, J). Thus, in CHILD males but not CHILD females, the severity of the BBB dysfunction was directly related to vasculature morphological changes within the ipsilateral cortex.

### Acute CHILD elicits a hyper-acute reduction in vessel complexity

**Figure 5:**
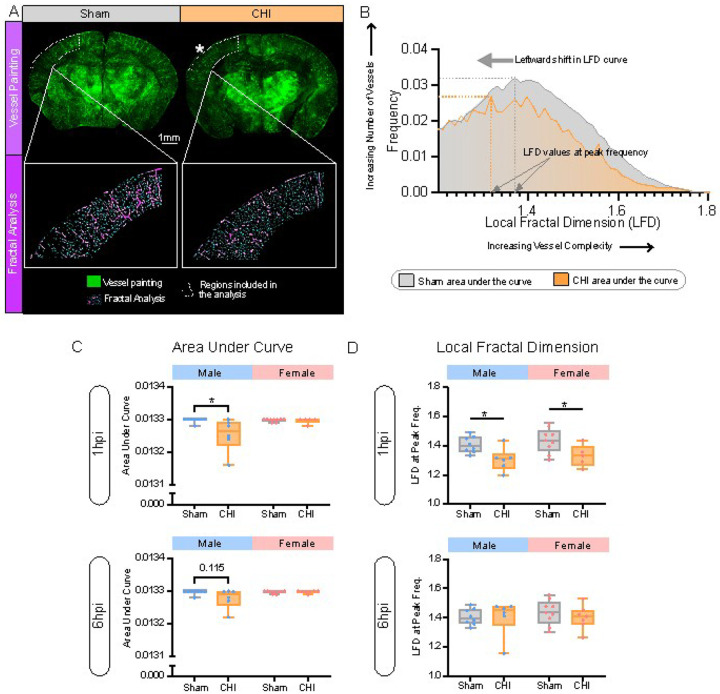
Cortical vascular complexity was reduced at acute epochs post-injury. **(A)** Representative vessel painted (VP) coronal sections from sham and CHILD mice with their corresponding fractal analysis images. White dotted outlines denote analysis regions. **(B)** Exemplar data from sham and CHILD mice for area under the curve and local fractal dimension (LFD) values at peak frequencies calculations derived from LFD histograms. CHILD resulted in a leftward shift in the average LFD histogram relative to shams, consistent with decreased vascular complexity. **(C)** Area under the LFD curve (AUC) measures for males and females at acute (1, 6hpi) reported a significant decreased AUC in CHILD males (*p≤0.05, unpaired t-test) but not in CHILD females. **(D)** LFD values at peak frequency were significantly reduced 1hpi (*p≤0.05, unpaired t-test) in males and females but not at 6hpi. (see Supplementary Figure 4 for additional time points). All bar graphs were represented as mean ±SD

A key hallmark of vascular damage is a reduction in vascular complexity which can be assessed using fractal measures [23]. Smaller vessels are potentially more vulnerable to mechanistic forces induced by the head rotation (Rodriguez-Grande, Glia 2018) and thus contribute to pathological progression of BBB breakdown. We assessed vascular complexity in coronal cortical vessels at the lesion site by generating fractal histograms (Fig. 5A, B; Supplementary Fig. 5). The resultant fractal histograms measures provide quantitative information about complexity (shift in LFD curve) and vessel numbers (area under the curve or AUC) (Fig. 5B). Early hyper-acute time points (1–6hpi) revealed a significant reduction in AUC at 1hpi in male CHILD mice compared with male shams (p = 0.031, unpaired t test), with no overt differences in female CHILD mice (Fig. 5C). At 6hpi in male CHILD mice the AUC started to recover with a trending significant reduction (p = 0.115, unpaired t-test) (Fig. 5C) with no significant differences were observed in either male or female CHILD mice at other time points up to 7dpi (Supplementary Fig. 6A, B).

Vessel complexity was assessed using the maximum local fractal dimension (LFD) at the peak frequency (Fig. 5B). At 1hpi, both CHILD male (p = 0.016, unpaired t test) and CHILD female (p = 0.037, unpaired t test) mice had significant reductions in LFD values consistent with reduced vessel complexity (Fig. 5B) but no differences in either male or female CHILD mice were observed at any other time points (Supplementary Figs. 4,5). These results further confirm that CHILD in male mice results in reduced brain vasculature and complexity at hyper-acute time points post-injury whereas CHILD in female mice elicited only decrements in complexity but not in vascular density.

### Presence of Immunoglobulin G (IgG) in CHILD mice signifies BBB breakdown

**Figure 6:**
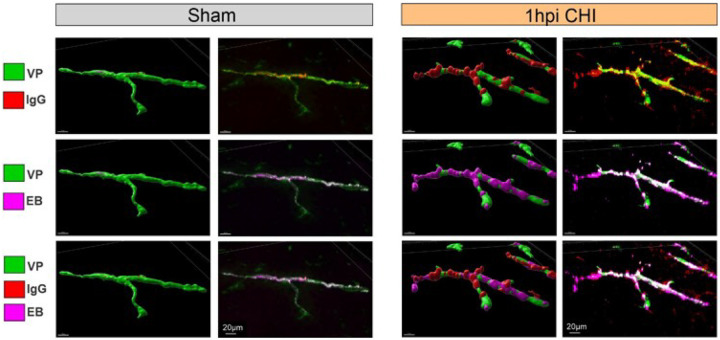
Extensive BBB disruptions are apparent in vascular reconstructions at 1hpi CHI. Representative 20X confocal images of ipsilateral cortex sections stained for IgG (red), vessel painting (VP, green), and Evans Blue (EB, purple), along with the corresponding 3D reconstructions. In sham mice (left panel), both IgG and EB remain confined within the vasculature. In contrast, in CHILD mice at 1hpi (right panel), there is extensive extravasation of IgG and EB from vessels in the ipsilateral cortex, confirming perivascular leakage.

Immunoglobulin G (IgG) extravasation in brain tissue after injury is a marker of BBB dysfunction as we previously described in CHILD at 1dpi (Rodriguez-Grande et al. 2018) and in adult CHI [26] and in juvenile TBI [11]. To further confirm BBB dysfunction, we undertook IgG staining at 1hpi when the most robust alterations in cortical vessels and EB extravasation were observed. Low magnification IgG-stained sections (Supplementary Fig. 6) were examined for representative cortical vessels that exhibited EB extravasation in male CHILD mice at 1hpi. Figure 6 illustrates the coincident labeling between vessels and IgG, vessels and EB and vessels, IgG and EB in sham mice (left panel) and CHILD mice (right panel). Sham mice did not exhibit any notable IgG or EB signals outside the vessels although in the merged images both are visible within the vessels themselves. In stark contrast, CHILD mice at 1hpi exhibited IgG extravasation staining protruding from vessels in discrete beads along injuried vessels (Fig. 6). Interestingly, extravasation EB staining presented larger coverage along the vessels than IgG staining. However, IgG staining was associated with EB-extravasation (Fig. 6), a confirmation of the vascular-BBB dysfunctions.

### Modeling the Interactions between Vascular Injury and BBB Dysfunction

**Figure 7:**
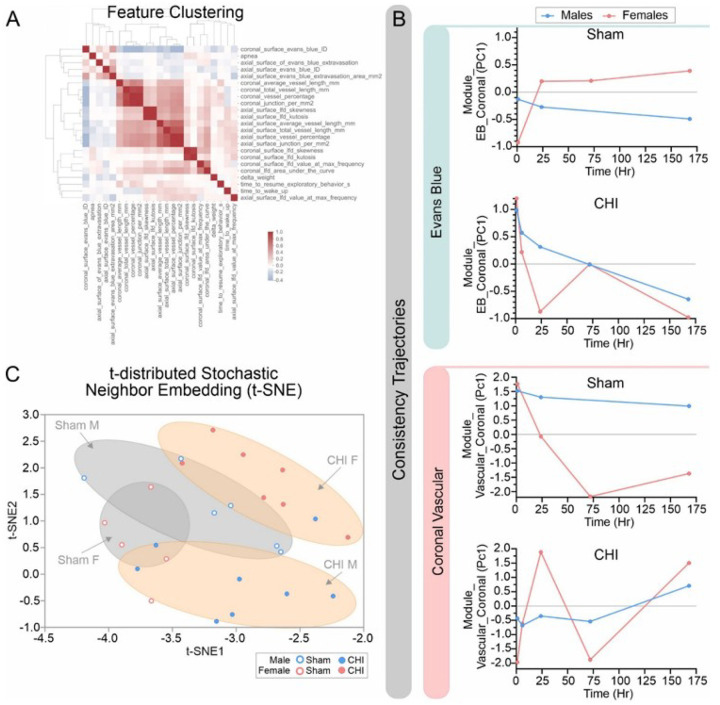
Vascular features strongly cluster in CHI mice. A) Clustering and heatmap representation illustrate the strong concordance between vascular features in CHI mice. B) Data reduction into feature related modules (see Supplemental Table 2) to model the temporal relationship. Consistency trajectories revealed that Evans Blue Leakage module exhibited poor consistency in sham mice while CHI mice showed high consistency at early time points. The Coronal Vascular Module sham male mice showed a stable trajectory while female sham mice were more variable. In contrast, CHI female mice showed no trajectory consistency whereas males had a relatively stable trajectory. C) To visualize the high dimensional phenotypes and their interactions in CHI and sham mice we employed t-distributed Stochastic Neighbor Embedding (t-SNE) from a set of the first principal component scores (PC1). As shown irrespective to time points, our vascular features were able to discriminate in a sex- and injury specific manner.

Given the wealth of the data acquired in this study we examined if modeling these vascular and BBB data could provide additional insights into the physiological mechanisms and the potential for predictive capabilities. The first step was to identify the correlation structure between all the variables (data) that were collected. Clustering of the data and its heatmap representation clearly illustrated that vascular measures strongly clustered together (Fig. 7A). Coronal vascular features, axial vascular features and complexity measures were all strongly aggregated. Physiological features (apnea duration etc.) exhibited a reduced clustering. Based on the clustering and heatmap analysis we undertook a data reduction approach whereby we consolidated groups of related features into modules. The final modules are summarized in Supplemental Table 2 and include Vascular Coronal, Vascular Axial, Leakage Evans Blue and Local Fractal Dimension (coronal and axial combined) and were utilized in subsequent analysis.

Modeling of temporal evolution of vascular and BBB disruption following brain injury are of importance, particularly in the context of patient management. Here we undertook trajectory analyses and coupled this to consistency measures to identify which features provide intuitive measures of biological coherence. We found that in the Evans Blue Leakage module sham mice (male or female) exhibited poor consistency values as might be expected as no CHI was induced (Fig. 7B, top panel). In contrast, the CHI mice showed high consistency at the earlier time points with decreasing consistency at later time points consistent with acute BBB disruption after brain injury (Fig. 7B, top panel). There were no overt sex differences. When we examined the coronal vascular module, male sham mice exhibited a stable trajectory while female mice had a more variable consistency (Fig. 7B, bottom panel). In male CHI mice there was a progressive increase in consistency in cortical vascular features that continued over 7dpi (Fig. 7B, bottom panel). The female CHI mice exhibited no consistency in these coronal vascular features until 1dpi that then precipitously declined by 3dpi with subsequent increased consistency by 7dpi, like that of male CHI mice (Fig. 7B, bottom panel). These consistency measures suggest that males after injury exhibited a more consistent trajectory of either BBB leakage or coronal vascular features than female CHI mice.

We now examined these multivariate relationships by employing t-distributed Stochastic Neighbor Embedding (t-SNE) to visualize these interactions. Specifically, we were interested in how injury and sex may jointly influence our highly dimensional phenotypes particularly in light of the heterogenous nature of concussion. These analyses highlighted sex and injury-specific clustering (Fig. 7C). While there was some overlap between sham male and female mice (as would be expected) the CHI male and female mice exhibited clear separations. As noted in our vascular and Evans Blue data and in the modeling above, male and female CHI mice clearly have unique features that allow separation.

## Discussion

Pediatric and juvenile mild traumatic brain injury (mTBI, concussions) and their subsequent pathologic sequelae are understudied. Most notably lacking is how blood-brain border (BBB) integrity is impacted by concussive injuries over time in clinic patients, as well as in rodent models. We used our CHILD mouse model utilizing a single impact at postnatal day 17 (PND17) reminiscent of pediatric concussion which includes a rotational component [15]. We describe here the distinct neurovascular and BBB trajectories in both male and female mice at hyper-acute and acute epochs after impact above the somatosensory cortex. Our key findings are: 1) weight reductions in CHILD mice, particularly males, 2) T2 values in ipsilateral cortical regions were reduced in CHILD males but increased in females, 3) hyper-acute EB extravasation in male and female CHILD mice, 4) hyper-acute decrements in vessel density that correlated with the presence of EB, but only in males but not female CHILD mice, 5) reductions in acute vessel complexity were only apparent in male CHILD mice, and 6) EB and IgG were visible adjacent to intracortical vessels in CHILD mice but not shams. We also demonstrate strong sex- and injury-specific relationships in our modeling approach. Taken together our results herein provide strong evidence for an early hyper-acute vascular and BBB disruption that is more prominent in males compared to female concussed juvenile mice. These early cerebrovascular perturbations may presage the subsequent development of long-term deficits that we have reported previously [19, 27].

### Changes in physiology and interference with brain development

Pediatric and juvenile brains are vulnerable to concussion given the rapid developmental growth of the brain and neuronal connectivity. Early physiological changes after concussion in early childhood have been described (i.e. appetite changes, cognition, mood etc. (see [28]). Sex differences in either concussion rates [29], neuroimaging features [30, 31] and acute and chronic outcomes [32] have suggested the existence of potential differences between males and females in both clinical and preclinical studies. In our juvenile model of concussion, male mice displayed a higher prevalence of transient apnea (44.44% of males) than females (24.32%), while both sexes experienced prolonged righting reflex times. The CHILD model is associated with rotational aspects at time of injury [15] and youth have been modeled to exhibit lower linear and rotational tolerance than adults [33]. Our rotational CHILD model can result in brain stem injuries, resulting in a transient disruption of the respiratory system function due to disturbance of medullary functions and temporary respiratory arrhythmia [34]. Righting reflexes in rodents are considered an alternative measurement of consciousness and alertness for determining TBI severity and prognosis [35] albeit in our study we found no overt sex differences. Thus, transient apnea and delayed righting reflex in the CHILD model represents a concussion landmark of the precipitating event compared to shams. We have previously described for the CHILD model a sex difference in recovery times which were shorter in males compared to female mice, in contrast to our current findings which had a larger number of replicates [14]. The severity of transient hypoxic events can represent a critical pathological landmark for concussion as the duration of hypoxia has been correlated to future long term cardiac dysfunctions in CHILD [18].

In the current study, reduced weight gain in CHILD mice was observed by 7dpi. While both sexes had reductions in weight gain, only male CHILD mice reported significant differences. Attenuated weight gain after repeated mild TBI has been described in rodents reflecting problems in pituitary and hypothalamic circuits, impeding normal growth and development [36, 37]. In a rat pup model of mild TBI, decrements in weight gain were coincident with growth hormone reductions during the acute period but were elevated chronically [38]. Such hormonal changes may also underlie progressive decreases in CHILD cerebrum volumes. Therefore, disturbance of hypothalamic function after CHILD may influence a host of physiological changes both acutely and long after the initial event.

### Early sex-dependent vascular alterations

Temporal neurovascular alterations, BBB dysfunction, vessel properties and morphology have been previously investigated in juvenile CHILD males where increased IgG staining in brain parenchyma has been described at 1dpi and resolved by 7dpi [15]. These alterations in BBB properties were associated with decreased neurovascular reactivity and decreased brain oxygenation peaking at 6hpi which then normalized [16]. These early functional vascular changes were then assessed 12 months post-concussion and CHILD male mice had increased numbers of capillary vessels [17]. Lacking from the literature is an assessment of neurovascular properties in male and female acutely after single pediatric concussion. This gap is critical considering known sex differences in youth after concussion injury [29].

We report significant reductions of pial and intracortical cerebral vessels for male CHILD mice at 1hpi. These decrements in cerebrovascular metrics were not significantly different in female CHILD mice (see Figs. 3, 4). Moreover, these sex-specific differences were also confirmed in our modeling (see Fig. 7). The hyper-acute reductions in vessel density, vessel total length and number of junctions normalized at later timepoints. Our current findings relied on the vessel painting method with perfusion of lipophilic dye through the vascular system [23]. One consideration is that mTBI may result in vascular hypoperfusion [39, 40] and reduced neurovascular coupling [16, 41], as previously reported. Vasculogenic processes are initiated within acute time windows with angiogenic molecules such as vascular endothelial growth factor (VEGF) being upregulated [42, 43]. While VEGF rapidly increases after TBI it is thought to require ~ 2 weeks for the complex molecular cascade to induce endothelial cell proliferation and migration, anastomosis, and glial cell recruitment [44]. Other vasculogenic pathways are also involved and in adult TBI activation of the Wnt/β-catenin pathway as early as 1dpi with increased Wnt5a levels at 7dpi where related to recovery of vessel density [45]. Our current study did not directly examine cerebral blood flow (CBF) at these acute time points, but T2-weighted imaging (specifically T2 relaxation) potentially sheds some additional light. We reported T2 relaxation was significantly decreased in male CHILD mice in cortical regions under or adjacent to the impact site but was significantly increased in female CHILD mice (Fig. 1F–I). Our current interpretation of these opposite sex effects is that in males there is a putative reduction in CBF and in metabolically active tissues results in increased oxygen extraction during transit. In females these changes are less pronounced acutely, and CBF may not be impacted to the same extent as in males. We have described such a mechanism for reduced T2 relaxation previously [46]. Therefore, multiple systems may contribute to the recovery of decreased vessel density, including recovery of perfusion and vasculogenesis.

Intracortical vascular complexity was also only altered in male CHlLD mice at 1hpi with no overt changes at later time points. In male CHILD mice the LFD at peak frequency was always increased (although not significantly) at each time point and normalized by 7dpi, which was not the case in female mice (Supplementary Fig. 4). While vascular complexity has not been extensively reported in TBI, we have noted in a cortical contusion injury model, initial loss of complexity that recovers with resumption of vessel density over time [23]. It is also important to highlight temporal differences between pial and intracortical vessels in our study, where we observed a longer duration of presumed recovery in intraparenchymal blood vessels (Fig. 4). It is well known that pial and intraparenchymal vessels present different anatomical, physiological and functional properties in healthy and diseased brain tissues [10, 47, 48]. These differences could suggest that in response to a mechanical injury, there is a potential greater vulnerability for intracortical compared to pial blood vessels to mechanical deformation. Our observations are strengthened by the importance of mechanical injury-induced cerebrovascular dysfunction that has been described clinically and in mouse models [49, 50].

### Sex-BBB alterations

In this study, BBB properties were assessed using EB injection 1hr prior to VP. Brief and early elevations of EB extravasation were observed for both sexes in CHILD mice, although only significant in females (Fig. 2B). These acute robust increases in EB leakage also exhibited high consistency in our trajectory modeling of both male and female CHI mice, which then declined with time post injury (Fig. 7B). Male CHILD mice exhibited an elevated EB extravasation at 1dpi, in concordance with our previous report [15], that then declined linearly to sham levels by 7dpi (Fig. 2C). This contrasts with the rapid decline at 1dpi of EB extravasation in female CHILD mice, further demonstrating sex differences in response to concussion and further supported in our trajectory modeling. Our histological demonstration of co-localization of EB and IgG outside of vascular structures in male CHILD mice, clearly demonstrates extravasation during the hyper-acute period after concussion. It is interesting to note that the IgG and EB staining do not consistently overlap and may be due to differences in molecular weights (EB: 0.9kDa, IgG: 150kDa). We and others have reported similar vascular IgG extravasation after concussion [51] and cortical contusion injury (CCI) [9]. Importantly, EB extravasation and VP-derived vessel metrics were significantly negatively correlated in CHILD male in both pial and intracortical assessments but not females (Figs. 3, 4). Decreased vessel density strongly correlated with larger EB extravasation. These results further support in male CHILD mice an acute BBB leakage, hypoperfusion and modified vessel angioarchitecture [16].

Changes in BBB after CHILD have been linked with early increased expression of the water channel aquaporin 4 (APQ4) at 1dpi with late developing astrogliosis (7dpi) (Rodriguez-Grande et al 2018). Even a mild mechanical stress on the brain produces significant vascular changes with decrease in flow and change in BBB properties, similarly to more severe TBI in pediatrics and adults [9, 20, 45, 52, 53]. The exact mechanism(s) behind these perturbations are still poorly understood and the pathophysiological vascular response differs over time. Mechanical forces due to linear and rotational acceleration can directly damage endothelial cell structures compromising junctional proteins that transition to BBB dysfunction and subsequent vasogenic edema [54–56]. Local changes in water homeostasis may compress nearby vessels and the presumed loss of perfusion observed in our results. Concurrently, endothelial cells in response to injury also release vasoconstrictors such as endothelin-1 (ET-1) [57, 58], further promoting vasoconstriction and altering vascular tone. Together, edema and ET-1-dependent vasoconstrictor activity could reduce CBF and may limit dye perfusion, consistent with the reduction observed in vessel painting and larger EB extravasation. These changes are resolved by 7dpi.

The molecular mechanisms involved in resolution of the BBB dysfunction post-injury are very poorly understood and understudied [7]. There are numerous putative proteins involved in BBB leakage, including caveolin-1 [59], which is expressed in the neurovascular unit and has been proposed as a key pathway in BBB recovery in juvenile moderate TBI [60]. Caveolin-1 expression after brain injury follows a similar timeline of the BBB changes and recovery in CHILD [60]. Other phenomena such as tight junction (TJ) loss after TBI in adult rodents leads to BBB loss of function acutely with subsequent restoration and reduced inflammatory activities [61]. Compromised BBB function allows entry of peripheral serum proteins (IgG), immune cells, and potential pathogens into the brain parenchyma, activating brain resident immune responses [62]. The heightened immune response, leading to microgliosis and astrogliosis, can further promote endothelial dysfunction. Thus, despite recovery from transient BBB permeability, the progression of secondary sequalae and the potential for chronic inflammation can lead to a compromised neurovascular unit [13]. The early neurovascular dysfunctions after CHILD may represent a critical event that leads to long-term neurovascular dysfunction and inflammatory sequelae observed in the CHILD mouse model of pediatric concussion [17–19, 63].

### Sex differences following pediatric concussion

A recent literature review found that 44% of animal TBI studies reported that females had better outcomes than males, while only 28% of the studies surveyed showed no sex differences [64]. Despite increasing studies using both males and females there is still a considerable gap about how sex modulates outcomes in pediatric concussion. It has been hypothesized that estrogen and progesterone play vascular protective roles in females by upregulating vasodilatory factors and reducing vasoconstrictive factors [64, 65], thereby improving microcirculation and vessel reactivity. Compared to males, females present with a lower prevalence of cardiovascular disease [66] and improved CBF after TBI [39, 67]. Adult female mice in a CCI model of TBI found acute (1dpi) increased astrogliosis and heme-oxygenase-1 expression (via estrogen) whereas male TBI mice had increased neovascularization via β-catenin [9]. However, sex hormones alone may not fully account for the differential vascular function, as sex differences in microvascular blood flow have been reported during infancy and adolescence [57, 67, 68].

Another potential contributor to sex differences in prepubertal juveniles is the differential expression of the predominant vasoconstrictor ET-1 in pediatric TBI, which is upregulated in males, but not in females [57]. The upregulation of ET-1 in males increases their susceptibility to cerebral autoregulatory impairments, leading to vasoconstriction and reduced perfusion. This potentially provides one mechanism for the reduced T2 values and reduction in vessel metrics we report here. Contrary to the males, the reduction of ET-1in females may be protective, thus explaining the lack of vasculature abnormalities despite pronounced BBB disruptions. Indeed, our trajectory and consistency modeling lends additional support related to strong sex differences, even at this early age. Our current study further reveals that sex differences in the vascular response to trauma are present and should incentivize further exploration into mechanisms underlying sex differences in juvenile TBI.

### Trajectory Modeling of Traumatic Brain Injury (TBI)

Nascent studies are now being to address the heterogenous nature of TBI recovery between groups and individuals, primarily focused on physiological, psychological and functional outcomes. Ren and colleagues, using trajectory analyses for emotive symptoms in a relatively small cohort of subjects, were able to discriminate between depression, anxiety and life satisfaction as well as within groups (low vs. high symptomology) [69]. Similarly functional motor and cognitive scores were able to differentiate recovery patterns in subjects with moderate to severe TBI [70]. A more comprehensive Track-TBI study with 2100 participants was able to identify seven unique trajectories based on recovery by using Glasgow Outcome Scale Extended (GOSE) [71]. These trajectories were also strongly associated with initial presentation of the GOS, computed tomography findings and psychiatric comorbidities. Other measures after TBI, such as patterns of sympathetic hyperactivity [72], executive function [73] and quality of life measures [74] also aggregate into distinct trajectories. To our knowledge, there are no clinical or preclinical studies examining trajectories related to vascular impairments and longer-term outcomes. While our study was confined to 7dpi, we demonstrate that trajectory modeling may have enhanced utility in discriminating between sex and injury subtypes bases on vascular and BBB leakage features. We suggest that future longitudinal studies utilize trajectory modeling to enhance our understanding of physiological and psychological outcomes after TBI.

### Limitations and future directions

There are several limitations and future directions related to our findings. Firstly, all the morphological and BBB leakage studies required sacrifice of the mice at discrete epochs. Continuous monitoring from the same animal for neurovascular function and BBB leakage using other techniques, such as MRI, would have provided additional temporal evolution insights. As noted above, the VP method can map the cerebrovascular networks, but only if they are being perfused. Correlation with other histological techniques (ie tomato lectin, etc.) could be applied. We also did not make any effort to disentangle the contribution of arteries and veins in concussion. Finally, there are number proteins responsible for modulating the BBB in health and disease, such as caveolins, claudins and others that likely play an important role not only in the acute injury phase but also during recovery; we did not explicitly assess those in our study. Future studies using imaging approaches such as MRI, in vivo confocal microscopy, miniscopes (as we have done [49]) or functional ultrasound imaging (fUSI) could and should be used to differentiate direct perfusion changes, particularly as they relate to early in life putative sex differences. Additional replicates or similar data from other published research could strengthen the impact of trajectory modeling.

## Conclusion

The novelty of our research is two-fold: 1) hyper-acute sex differences in a clinically translatable model of juvenile mTBI (CHILD) that exhibits acute, chronic and long-term (12 months) pathophysiology [17, 19, 63], and 2) hyper-acute vessel changes that strongly correlate with BBB leakage in a sex-dependent manner. Our findings demonstrate that CHILD induces an early acute neurovascular change with loss of vascular density and BBB dysfunction transiently, recovering by 7dpi only in males and not females. We posit that these hyper-acute changes within the neurovascular unit primes the injured brain to contribute to the long-term cellular, molecular, neurovascular and behavioral perturbations we, and others have observed at 12–18 months post-concussion [17, 19, 63]. The presence of sex differences during the prepubertal period highlights the importance of incorporating biological sex as a determinant of injury response in future mechanistic and therapeutic pediatric mTBI studies.

## Supplementary Material

This is a list of supplementary files associated with this preprint. Click to download.

- Cav1CHIBBBSupplementalFigures011926ao.docx

## Data Availability

All protocols and data supporting the findings are available upon request to the corresponding authors.

## Abbreviations

AQP4: Aquaporin 4
BBB: Blood-brain border
CCI: Cortical contusion Injury
CHI: Closed head injury
CHILD: Closed head injury with long term disorders
DiO: 3,3’-dioctadecyloxacarbocyanine
dpi: Days post-injury
EB: Evans blue
ET-1: Endothelin-1
hpi: Hours post-injury
IHC: Immunohistochemistry
LFD: Local fractal dimension
MMP: Matrix metalloproteinase
MRI: Magnetic resonance imaging
ms: milliseconds
mTBI: Mild traumatic brain injury
PBS: Phosphate-buffered saline
PCA: Principal component analysis
PFA: Paraformaldehyde
PND: Postnatal day
ROI: Region of interest
ROS: Reactive oxygen species
SWI: Susceptibility-weighted imaging
T2: T2-weighted
TBI: Traumatic Brain injury
TJ: Tight junction
t-SNE: t-distributed Stochastic Neighbor Embedding
VEGF: Vascular endothelial growth factor
